# Proven Anti-Wetting Properties of Molybdenum Tested for High-Temperature Corrosion-Resistance with Potential Application in the Aluminum Industry

**DOI:** 10.3390/ma14185355

**Published:** 2021-09-16

**Authors:** François Gitzhofer, James Aluha, Pierre-Olivier Langlois, Faranak Barandehfard, Thabang A. Ntho, Nicolas Abatzoglou

**Affiliations:** 1 Department of Chemical & Biotechnological Engineering, Université de Sherbrooke, Québec, QC J1K 2R1, Canada; james.aluha@usherbrooke.ca (J.A.); pierre-olivier.langlois2@usherbrooke.ca (P.-O.L.); faranak.barandehfard@usherbrooke.ca (F.B.); nicolas.abatzoglou@usherbrooke.ca (N.A.); 2 Pyrotek Inc., Centre de Recherche & Développement, 4125 Rue de la Garlock, Sherbrooke, QC J1L 1W9, Canada; 3 Cerdo Trading (Pty) Ltd., 05 Basalt, 15 Kapital Street, Northriding, Johannesburg 2162, South Africa; thabang.ntho123@gmail.com

**Keywords:** ALCAN immersion test, anti-wetting, Mo, Al-Mg alloy, intergranular corrosion

## Abstract

The behavior of Mo in contact with molten Al was modelled by classical molecular dynamics (CMD) simulation of a pure Mo solid in contact with molten Al at 1200 K using the Materials Studio^®^. Results showed that no reaction or cross diffusion of atoms occurs at the Mo(s)–Al(l) interface, and that molten Al atoms exhibit an epitaxial alignment with the exposed solid Mo crystal morphology. Furthermore, the two phases {Mo(s) and Al(l)} are predicted to interact with weak van der Waals forces and give interfacial energy of about 203 mJ/m^2^. Surface energy measurements by the sessile drop experiment using the van Oss–Chaudhury–Good (VCG) theory established a Mo(s)–Al(l) interface energy equivalent to 54 mJ/m^2^, which supports the weak van der Waals interaction. The corrosion resistance of a high purity (99.97%) Mo block was then tested in a molten alloy of 5% Mg mixed in Al (Al-5 wt.%Mg) at 1123 K for 96 h, using the ALCAN’s standard “immersion” test, and the results are presented. No Mo was found to be dissolved in the molten Al-Mg alloy. However, a 20% mass loss in the Mo block was due to intergranular corrosion scissoring the Mo block in the ALCAN test, but not as a result of the reaction of pure Mo with the molten Al-Mg alloy. It was observed that the Al-Mg alloy did not stick to the Mo block.

## 1. Introduction

Equipment failure caused by corrosion wear, which is accelerated by contact with molten aluminum and its alloys, is one of the main problems facing the Al industry [1]. Various investigations into the resistance behavior of countless materials, including metals, Fe-based alloys, and their corresponding apparatus for high-temperature applications have been conducted for corrosion wear in molten Al and its alloys. Since Al has a tendency to stick to other metals, a coating of boron nitride, for example, is spray-painted to cover sow moulds in order to prevent the molten Al from sticking [2]. However, the process becomes tedious because this coating must be done every time. Therefore, a permanent solution would be either to add a mould-release agent, which can generate such a coating for moulds that are subjected to a liquid or flowable Al material, or create a method for producing such a coating [3]. In the Al industry, which produces Al-Mg alloys, corrosion leads to contamination of the alloy products arising from the solid impurities that come from, for example, the sow molds, rotor material, or degraded refractories [4]. 

Using bulk corrosion-resistant materials sometimes comes at a price, and therefore, one of the effective ways of reducing cost is to apply an expensive coating as a thin film on a cheaper material. Some researchers have produced pure Mo coatings on steel via thermal spraying and tested them for corrosion resistance [5]. Anti-corrosion coatings include those that create barriers between the substrate materials and the corrosive environments, those that catalytically inhibit the corrosion processes, and those coatings that act as sacrificial materials [6]. By studying the thermodynamics of these materials, one can show how conditions may be adjusted to make corrosion impossible.

Normally, the corrosion process begins with the wetting of the solid material by the molten Al liquid as a result of high adhesive forces between them. This is followed by a reaction that creates an interface with a different chemical composition (with a strong effect on the magnitude of the interfacial free energy, and subsequent changes in the contact angle), and if this interfacial material is highly soluble, cracks are opened, more Al liquid penetrates into the material, thus exacerbating the corrosion process [4]. We are therefore proposing the use of a plasma-sprayed coating with non-wetting characteristics towards molten Al and Al-Mg alloys. Besides the chemistry of the materials that are in contact, some of the factors that exacerbate the wetting phenomenon include high surface roughness, sample porosity, and chemical reactions that lead to a low contact angle with the molten Al [7]. 

According to ASM International (2000) classification [8], about ten categories of wet (or aqueous) corrosion exists in metals, that is, (i) uniform or general corrosion, (ii) galvanic corrosion, (iii) pitting corrosion, (iv) crevice corrosion, which includes corrosion under tubercles or deposits, filiform corrosion, and poultice corrosion, (v) erosion-corrosion, including cavitation erosion, (vi) exfoliation, (vii) fretting corrosion, (viii) intergranular corrosion, (ix) de-alloying, and (x) environmentally assisted processes such as cracking, including stress cracking corrosion, corrosion fatigue, and hydrogen damage [6]. It has been advanced that corrosion in the Al-Mg melt (with ±4% Mg) begins with the initial formation of MgO, and since MgO is porous, it allows the penetration of oxygen from air into liquid Al and acts as a catalyst in the nucleation of Al grains [9]. 

**Figure 1:**
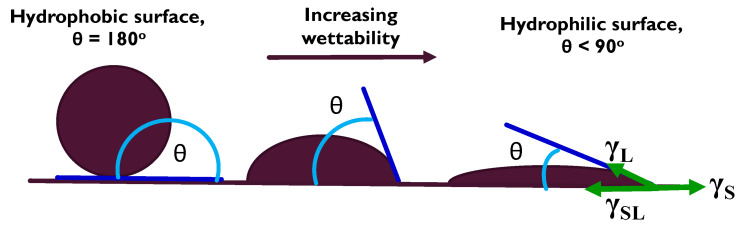
The wettability of a solid surface.

Ceramics such as aluminosilicate refractories, Al_2_O_3_, AlN, SiAlON’s, Si_3_N_4_, and graphite are characterized as inert in molten Al and its alloys. The corrosion resistance of metals is generally poorer than that of inert ceramics, although the durability of some metals such as Ti and Nb is relatively good [10]. Mo shows a low solubility in molten Al [11], while the addition of Mo improves the corrosion resistance of some alloys in molten Al at 750 °C [12]. Additionally, where Mo-rich islands exist in a material, they have been found to strengthen its matrix and increase its hardenability [13], with Ti-Mo alloys as a good example [14], while PGMs (Ru and Os) that have the HCP structure create a significant hardening effect (with for example, Pt) to form a practically unworkable material due to their extreme hardness [15]. Therefore, factors that influence the durability of a material in molten Al include how vigorous the agitation is, the presence of surface coatings, and their grain size, with grain boundaries being the point of weakness in the infiltration of molten Al. This is in addition to the dynamics of interfacial layers that influence the wettability of the solid material. Figure 1 illustrates the wettability of a solid surface as a function of its contact angle with the wetting liquid [16].

The wettability of the solid surface is determined by the Young Equation (1):

(1) $$\gamma_{S}=\;\gamma_{SL}+\gamma_{L}\cos\theta$$

where *γ_S_* is the solid surface energy, *γ_L_* is the liquid–vapor interfacial energy (surface tension), and *γ_SL_* is the solid–liquid interfacial energy.

Today, the wetting phenomenon between molten metal and various materials is receiving considerable attention. In some studies involving molten Fe on a graphite substrate, it was observed that the work of adhesion declines by about 5 times with increasing temperature in the range of 1250–1500 °C, which is indicative of a chemical interaction that creates structural changes in the molten metal [17]. In order to improve the anti-stick properties of coating materials used in Al industry, the material of choice should have low surface energy, or conversely, its contact angle with molten Al must be increased. 

Therefore, the objective of this research is to establish the anti-wetting properties of Mo metal as a potential coating material that can be used in thin metallic films applicable to the Al industry. For a start, we modelled and tested bulk Mo in molten Al to prove its anti-wetting capability. Since Mg is regarded as a surface-active element, it was added to the molten Al to enhance wettability [18], as a material surviving such aggressive Al-Mg media implies that it could make a Mo-based coating worthy of consideration. 

The hypothesis being tested here was to determine whether the pure Mo metal without any impurities or other inclusions could present irrefutable evidence for anti-wetting properties when in contact with molten Al alloys. The aim of investigation was to predict these characteristics through ab initio modelling and experimentally test and validate the potential of pure Mo metal to exhibit strong corrosion resistance properties towards a molten Al-Mg alloy. The novelty in this work is hereby defined by a combination of the following three fundamental considerations:

- The use of modelling to predict and demonstrate the anti-wetting properties of bulk Mo in contact with molten Al;
- Provide experimental proof of the anti-stick properties of metallic Mo in a molten Al alloy using the ALCAN standard immersion test, thus leading to a potential application in the Al industry;
- The application of radio frequency (RF) suspension plasma-spray (SPS) technology in synthesizing high-density Mo-based coatings, a process that cannot be easily achieved due to Mo’s high melting point at 2896 K (2623 °C).

Details of the design and optimization procedures of the Mo-based coatings, providing the plasma synthesis parameters, followed by extensive characterization and testing of the materials thereof are beyond the scope of this article, and these issues will be tackled separately. Nonetheless, the purpose of this article is exclusively to justify the possibility of synthesizing and applying such promising coatings in the Al industry. 

## 2. Materials and Methods

### 2.1. Modelling as a Predictive Tool

**Figure 2:**
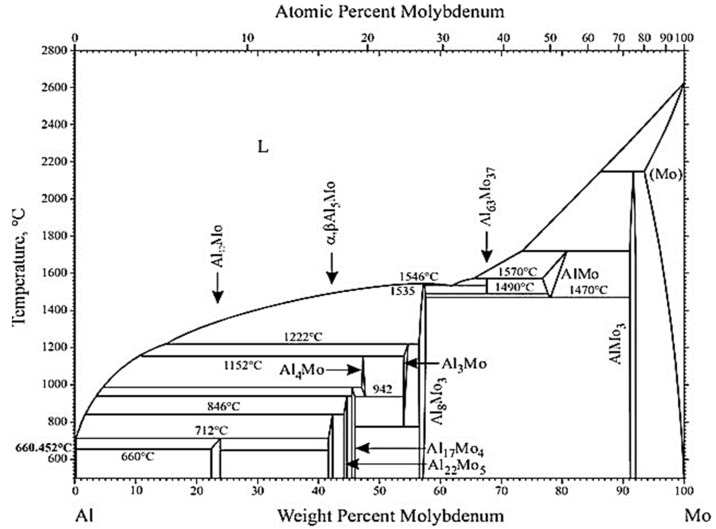
The Al-Mo phase diagram by Okamoto [19].

The binary Al-Mo phase diagram in Figure 2 indicates the high probability of creating numerous Al-Mo phases when pure Mo makes contact with Al metal at high temperatures [19]. Therefore, analysis of the corrosion products between Al and Mo can be a complex issue to deal with since the product composition also varies with temperature and may change with contact time along the way. For example, it has been asserted that at the Al–Mo interfaces, there are many possibilities of forming Al-Mo alloys depending on the temperature, and may include Mo_3_Al_8_, Mo_4_Al_17_, Mo_5_Al_22_, and MoAl_5_ at 735 and 785 °C (1008 and 1058 K), while at 835 °C (1108 K), the Mo_3_Al_8_, Mo_4_Al_17_, and Mo_5_Al_22_ phases may be observed, and the Mo_3_Al_8_, MoAl_4_, and Mo_4_Al_17_ phases could exist at 915 °C (1188 K) [10].

#### 2.1.1. Simulation by FactSage™

Some preliminary modelling was performed using FactSage™ thermochemical software version 7.2 (Montreal, QC, Canada) [20], in order to confirm the alloying properties of bulk Mo when it comes into contact with a molten Al-Mg alloy in the range of the experimental temperature (1123 K).

#### 2.1.2. Simulation Using Classical Molecular Dynamics

In this study, models involving the interaction of molten pure Al with solid Mo metal were developed from the first principles, employing the Materials Studio^®^ package (BIOVIA Dassault Systèmes, Vélizy-Villacoublay, France) [21]. Since rapid developments in materials research require an efficient way of constructing accurate and transferable interatomic potentials, the embedded-atom method (EAM) with potentials based on density functional theory (DFT) was used because it is a widely acceptable method to generate the semi-empirical interatomic potentials [22]. EAM is practical enough for calculations involving defects, surfaces, and impurities in metals [23], giving a reasonable description of metallic cohesion and ground-state impurity, whose energies and the potential of a short-ranged pair (of first neighbors only) are sufficient enough to fit the main properties of the bulk crystal. Using EAM, the total energy (E) of the crystal can be expressed through Equation (2) as follows:

(2) $$E=\;\frac{1}{2}\underset{i,j,\;\;i\neq j}{\sum }\emptyset_{ij}\left(r_{ij}\right)+\;\underset{i}{\sum }F_{i}\left(\rho_{i}\right)\;$$

where Ø*_ij_* represents the pair energy between atoms *i* and *j* separated by a distance *r_ij_*, and *F_i_* is the embedding energy associated with embedding an atom *i* into a local site with an electron density, *ρ_i_*. The electron density *f_j_(r_ij_)* at the site of atom *i* arising from atom *j* at a distance of *r_ij_* can be calculated using Equation (3) as follows:

(3) $$\rho_{i}=\;\underset{j,j\neq i}{\sum }f_{j}\left(r_{ij}\right)\;\;\;\;\;\;\;\;\;\;$$

To simulate the interaction and behavior of materials in a molten Al environment, the first step is to find a suitable force field that can accurately reproduce the physical macro-properties of liquid Al. This means that the applied force field should predict, as close to experimental values as possible, the density and melting temperature of Al metal to begin with. In this study, the EAM potential database reported in the literature [24] was used to generate the interatomic potentials of both Al and Mo for the simulation of the interaction between metallic Mo with liquid Al at 1200 K. 

In order to determine the melting point of a material, the unit cell of a solid is heated until it melts, and during modelling, this requires running the constant-volume constant-temperature ensemble (NVT), which is also referred to as the canonical ensemble. Otherwise, one can run the constant-temperature–constant-pressure ensemble (NPT), while escalating the thermostat temperature throughout the run, in a way mimicking a real experiment. However, due to the short time scales involved in molecular dynamics (MD), the rate of heating is very high. This coupled with the small system sizes results in super-heating, thereby providing only an upper boundary to the melting temperature. Similarly, a liquid may also be cooled until it crystallizes, giving hysteresis bounds between which the melting temperature may be found. 

A slightly more sophisticated procedure is the coexistence method, where a solid–liquid interface cell is constructed at a temperature near the predicted melting temperature. The main calculation in this approach is performed in the constant-energy-volume ensemble (NVE), also known as the microcanonical ensemble, or in the constant-pressure-enthalpy ensemble (NPH), which is the analogue of the NVE ensemble, where the size of the unit cell is allowed to vary [25]. If the temperature of the system as a whole lies below the melting temperature, a certain region of the cell will crystallize, thereby generating latent heat and raising the temperature. Likewise, if the temperature is too high, some of the cells will melt and cool the system, and in this way, if MD calculations are run long enough, they will equilibrate to the melting temperature. Therefore, in this work, the coexistence method was used within the NPH ensemble to predict the melting point of Al metal.

To build the coexistence model of liquid and solid Al, a supercell of Al (100) consisting of 32,000 atoms was created. To reduce stress, an MD simulation was run at 800 K for a total simulation time of 100 ps using the Velocity Scale thermostat in the NVT ensemble. All the calculations were done at an integration time step of 10 fs, large enough to cover a huge part of the configuration phase space without introducing numerical instability. The lattice parameters for this force field were equilibrated in the NPT ensemble using the Nosé–Hoover–Langevin (NHL) thermostat [26], and the Parrinello–Rahman barostat, permitting change in the cell shape and both pressure and stress to be controlled. As with temperature, the pressure (and stress) control mechanism must produce the correct statistical ensemble. This means that the probability of a certain configuration occurring must obey the laws of statistical mechanics.

A pressure of one atmospheric unit was applied in all NPT and NPH calculations with the resulting model being solid Al near the melting point. Similar calculations were repeated to build a model of liquid Al, except that the simulations were run at 1200 K. The predicted liquid-Al density was then used to build a liquid–solid model for the coexistence method simulation to determine the melting point using the NPH ensemble to equilibrate the system at the melting temperature predicted by the force field. A detailed description of this method is available in the literature [27], in addition to a good overview of the different methods of determining melting points [28].

All calculations reported here were carried out using the Forcite module within the Materials Studio^®^ package [21], using a 3-D model of metallic Mo that is built to interact with molten Al at 1200 K. Starting from a Mo (100) unit cell, a 25 × 25 × 25 supercell was created, resulting in a cubic model with a lattice parameter of 78.67 Å and a total of 31,250 atoms. Model stress was reduced using the Velocity Scale thermostat in the NVT ensemble at 800 K with an integration time step of 10 fs over a total simulation time of 100 ps. While keeping the same simulation time and integration time step, the lattice parameter was equilibrated in the NPT ensemble using the NHL thermostat and Anderson barostat at an applied pressure of one atmospheric unit (101.325 kPa). After achieving convergence, the resulting model was used to build a coexistence model of solid Mo with liquid Al (Al_25128_Mo_31250_)—using the predicted density of Al at 1200 K. The steps described above were repeated in order to reduce stress and to equilibrate the c lattice parameter of the Mo_(s)_–Al_(l)_ model at 1200 K over a total simulation time of 1000 ps (100,000 steps).

### 2.2. Surface Energy Determination

The wettability of the Mo samples was studied using the Krüss Advance goniometer model DSA25E (KRÜSS, Hamburg, Germany). Since the contact angle, surface free energy, and adhesion are related, the wettability of a surface can be estimated by determining the contact angle between a liquid and a solid. Considering the influence polar and dispersive surface free energy components have on wetting and adhesion, a set of three different liquids are used to determine the contact angles of the solid surface with the test liquids. For example, some authors have used distilled water, diiodomethane, and ethylene glycol at surface temperatures in the range of 20–120 °C (293–393 K) [29]. By applying the sessile drop method, which uses drop-shape analysis as the standard arrangement for the optical measurement of the contact angle, the contact area between the liquid and the solid must remain constant during data acquisition. 

In order to predict or mimic the interaction energy between liquid Al and the bulk Mo surface, the van Oss–Chaudhury–Good (VCG) theory was applied [30], represented by Equations (4) and (5). Since surface energy is dependent on the interaction of the solid material with any three liquids, diiodomethane (CH_2_I_2_) was used as the non-polar liquid, while water (H_2_O) and formamide (NH_2_CHO) were used as the polar liquids [31], though surface energy has been found to increase slightly with temperature [32]. In this technique, which works best for inorganic surfaces [33], the contact angle measured at 25 °C (298 K) was utilized to calculate the surface energy of our samples.

(4) $$\left(1+\cos\theta \right)\gamma_{L}=2\left(\sqrt{\gamma_{S}^{LW}\gamma_{L}^{LW}}+\sqrt{\gamma_{S}^{+}\gamma_{L}^{-}}+\sqrt{\gamma_{S}^{-}\gamma_{L}^{+}}\right)\;\;\;\;\;\;\;\;\;$$

(5) $$\gamma_{S}=\gamma_{S}^{LW}+\gamma_{S}^{AB}=\gamma_{S}^{LW}+2\sqrt{\gamma_{S}^{+}\gamma_{S}^{-}}\;\;\;\;\;\;\;\;\;\;\;$$

where *θ* is the contact angle between the liquid and the solid; *γ_L_* and *γ_S_* are the surface tensions of the liquid and the solid, respectively; $\gamma_{L}^{LW}$and $\gamma_{S}^{LW}$are the apolar or Lifshitz–van der Waals (LW) interactions; and $\gamma_{S}^{AB}$, $\gamma_{L}^{+}\gamma_{S}^{-}$, and $\gamma_{S}^{+}\gamma_{L}^{-}$ are polar or Lewis acid–base (AB) interactions for the liquid (L) and the solid (S). 

### 2.3. The ALCAN Standard Immersion Test

The ALCAN standard ‘immersion’ test involves submerging a sizeable block of material in molten Al for 96 h. Various approaches have been used to perform the ALCAN standard test, which include the ALCAN Cup Test and various other forms of the Static Immersion Test or the Dynamic Immersion Test [34]. In this investigation, a Mo-metal block of high purity (99.97%, supplied by Plansee, LLC, Franklin, MA, USA) with mass of 396.3 g was kept in a clay-bonded graphite crucible with 2 kg of Al alloy 5082 at room temperature. The crucible was placed in an electric furnace and heated at a rate of 2 °C·min^−1^ to 850 °C (1123 K) [35]. In order to overcome the loss of Mg in the alloy during the test due to evaporation, 24 h after initiating the test, 40 g of Mg was added daily to maintain the concentration of 5% Mg in the alloy [36]. After cooling, the excess Al alloy sticking to the samples was removed from the sample using concentrated hydrochloric acid (12 M) for a maximum of 10 min and then rinsed with water, after which the sample was kept in an oven at 110 °C (383 K) for a minimum of 1 h.

### 2.4. Materials Characterization

#### 2.4.1. Optical Microscopy 

Through imaging, the Keyence VHX-5000 optic microscope (Keyence Corporation of America, Itasca, IL, USA) was used to monitor the anti-wetting properties and the corrosion resistance of the pure Mo-block in contact with the molten Al-Mg alloy during the ALCAN immersion test.

#### 2.4.2. Elemental Analysis by Optical Emission Spectrometry (OES) 

The SpectroMaxx stationary metal analyzer (from AMETEK Inc., Berwyn, PA, USA), which is mainly used for testing materials in the metal industry, was employed for the elemental analysis of the molten Al-Mg alloy samples. A sample of the fresh Al-Mg alloy was scooped from the crucible before and after the ALCAN immersion test to determine how much Mo had dissolved in the alloy after 96 h. In principle, analysis by OES uses atomic emission spectroscopy and is the ideal method of elemental metal analysis since all the elements present in the sample are normally analyzed directly and simultaneously. 

#### 2.4.3. Scanning Electron Microscopy (SEM)

Samples were analyzed before and after the ALCAN immersion test on a Hitachi S-4700 SEM Field-Emission Scanning Electron Microscope (Tokyo, Japan), equipped with an energy dispersive X-ray (EDX) X-Max Oxford spectrometer (Abingdon, UK), capturing both secondary and backscattered electron images (SEI and BEI) operated at 20 kV [4]. EDX analysis for elemental composition and distribution in the samples with subsequent X-ray elemental mapping was derived from the SEM imaging. 

#### 2.4.4. X-ray Diffraction (XRD)

Phase analysis of the materials by X-ray diffraction (XRD) was conducted on a PANalytical Philips Panalytical X’pert Pro-MPD X-ray diffractometer (Almelo, The Netherlands), set in the Bragg–Brentano configuration with proportional Xe point detector. The instrument is fitted with Ni-filters for the Cu Kα radiation produced at 40 kV and 50 mA, wavelength alpha1 (λ = 1.540598 Å), and was operated on factory-installed Analytical Data Collector software in the 2θ-angle range of 20–105°, at a scanning speed of 0.04° [2θ]-angle per min, a step size of 0.02°, and a step time of 0.5 s. Data analysis was done using Materials Data Inc. software: The MDI JADE 2010 (version 7.8.4 @2020-04-23) and the collected data compared with the Powder Diffraction Files in the Database (version 4.2001) using the PDF-4+ software 2018 (version 4.20.0.1), published by the International Centre for Diffraction Data (ICDD).

## 3. Results

The findings in the current investigation are divided into two parts:

(a) Computer simulation results, which indicate that modelling as a predictive tool using both FactSage™ thermochemical software and Classical Molecular Dynamics showed that there would be no reaction and atomic diffusion at the interface between the Mo block and the molten Al alloy. The estimation of the surface energy by the VCG theory using the sessile drop experiments equally predicted a weak surface interaction at the Mo(s)–Al(l) interface.
(b) Experimental data from the static ALCAN immersion test agree with the simulation results, although some traces of Al-Mo alloys were detected on the Al-rich side of the Mo(s)–Al(l) interface. A weak interaction existed between the Al-Mo alloys and the solid Mo block (Mo-rich side), making it easy for Mo to peel off and demonstrate its anti-wetting properties; we suspect that the 20% mass loss on Mo was due to the chemical attack along the grain boundaries leading to intergranular corrosion.

### 3.1. Computer Modelling

#### 3.1.1. Simulation by FactSage™ 

**Figure 3:**
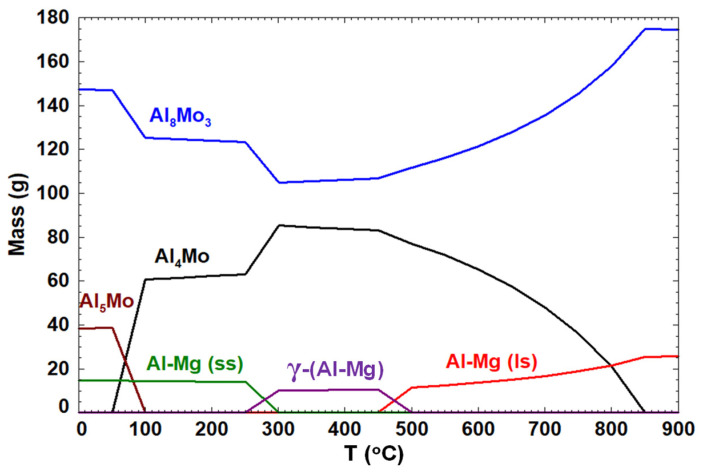
A FactSage™ prediction of the anti-wetting properties of solid metallic Mo in contact with molten Al-5 wt.%Mg alloy, with Al-Mg(ss) as a solid solution and Al-Mg(ls) as a liquid solution.

At the interface between pure Mo and the molten Al-Mg alloy, it was predicted using FactSage™ modelling that a reaction between Mo and Al is likely to occur to produce several binary alloys (Al_4_Mo, Al_5_Mo, and Al_8_Mo_3_) as portrayed in Figure 3. The prevailing phase at 1123 K (850 °C), the temperature of the ALCAN immersion test, is the Al_8_Mo_3_ alloy.

#### 3.1.2. Simulation by Classical Molecular Dynamics 

As a validation tool, the density of Al at 1200 K was used as an input in building the coexistence models in order to determine the melting point of Al as well as the interaction of metallic Mo with liquid Al. The Anderson barostat was used to keep the initial model in the cubic shape, and Table 1 provides the coexistence model lattice parameters of Al before and after melting. 

**Figure 4:**
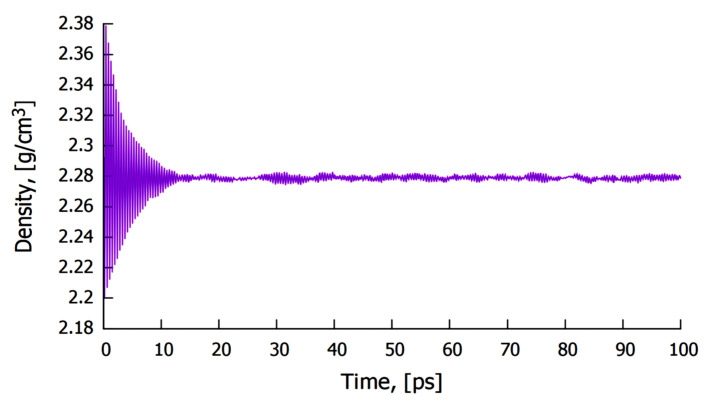
The predicted average density of molten Al at 1200 K.

At an equilibrium lattice parameter of 85.69 Å, with a total of 32,000 atoms, the average density of molten Al at 1200 K was predicted to be 2.279 g/cm^3^ as shown in Figure 4. This value compares well with reported experimental results of the density of Al [37], and those reported in literature at this temperature [38]. The predicted density of Mo at 1200 K by the same model was found to be 10.19 g/cm^3^, which compares well with the data value of 10.22 g/cm^3^ at ambient conditions.

**Figure 5:**
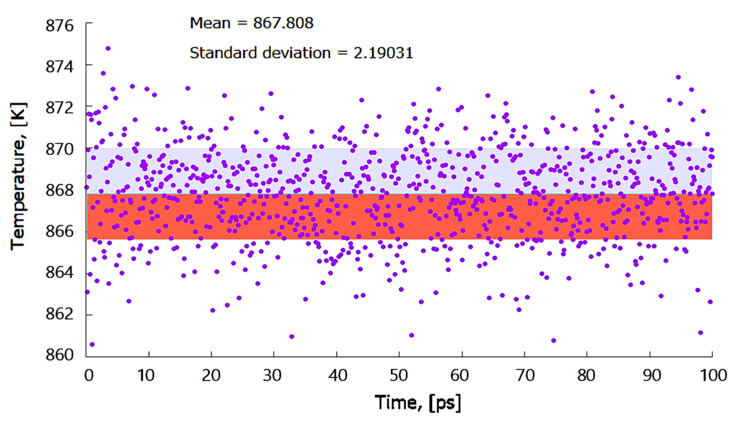
The predicted mean melting point ™ of Al using the coexistence method.

The efficacy of using a particular force field to simulate properties of molten materials requires one to test the accuracy of such a force field in predicting a melting point temperature of a material (*T*_m_) as close as possible to experimental results. With molten Al being of significant interest in this research, the suitability of using the modified embedded-atom method (MEAM) potential [27] was preferred. The coexistence model was built and equilibrated at 800 K using the isothermal-isobaric (NPT) ensemble before equilibrating the whole system using the isoenthalpic-isobaric ensemble (NPH) to determine *T*_m_. The model predicted the mean *T*_m_ value of Al (100) as 868 K as shown in Figure 5 and underestimated the experimental value by about 65 K (7%). This error margin is still acceptable considering that it is an approximation that shows potential for application to model systems that involve molten Al metal. 

Since simulation times applied in most studies are normally shortened, these results could possibly be improved to get closer to the experimental value by running longer simulations to allow for the full evolution of the system. The mean interaction surface energy at the interfaces was defined by Equation (6):

(6) $$E_{int}=\;\frac{\left(E_{layer1}+\;E_{layer2}\right)-\;E_{total}\;}{2*A}\;\;\;\;\;\;\;\;\;$$

where *E_int_* is the interaction energy between the two layers, *E_total_* is the total surface energy at the interface, *E_layer_*_1_ is the individual surface energy of layer 1 (Al), and *E_layer_*_2_ is the individual surface energy of layer 2 (Mo), while A is the unit surface area (Å^2^).

**Figure 6:**
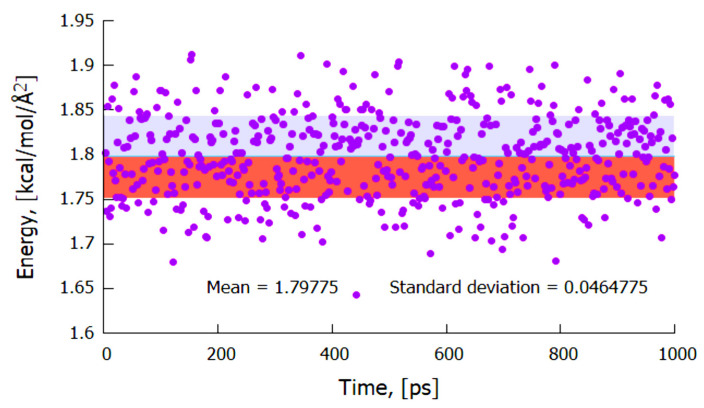
The mean interfacial energy between Mo (100) and Al (100) was 1.8 kcal/mol normalized to surface area (predicted by the coexistence method of molten Al at 1200 K).

The simulated mean interaction surface energy between two pure bulk Mo-Mo (100) slabs at 1200 K was found to be 13.67 kcal/mol (normalized per unit surface area in Å), and assuming that the atomic radius of Mo = 1.4 Å, this is equivalent to 1542 mJ/m^2^ and indicates strong metal–metal bonds. Furthermore, the predicted mean interfacial energy between the solid Mo (100) and ‘liquid’ Al (100) surfaces using the coexistence method for molten Al at 1200 K was found to be 1.8 kcal/mol/Å^2^ (~203 mJ/m^2^), as shown in Figure 6. This alludes to a weak van der Waals interaction between Mo and molten Al.

**Figure 7:**
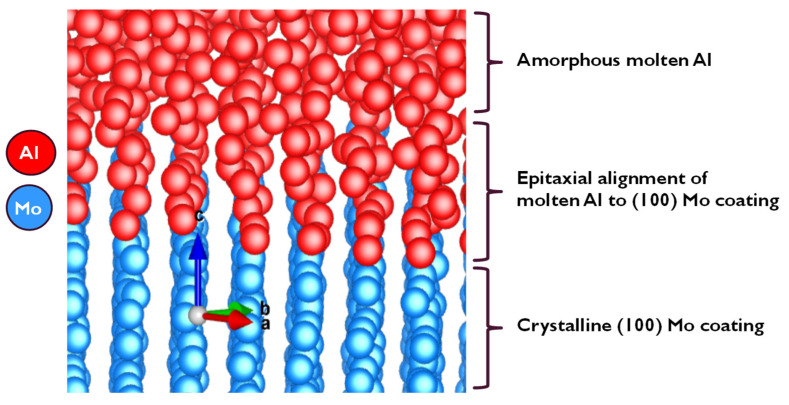
A model depicting the anti-wetting properties of solid metallic Mo in contact with molten Al at 1200 K.

Figure 7 portrays an output model between the metallic Mo (100) layer and the molten Al (100), where the molten Al atoms are seen to align and mimic the crystal structure of the Mo atoms at the interface. Since the Mo crystal lattice is BCC while that of Al is FCC, a considerable mismatch would be expected when they come into contact with each other. However, after equilibration at 1200 K, and based on a classical MD simulation of the Mo_(s)_–Al_(l)_ model with a trajectory that used a total simulation of over 1000 ps, three important features were predicted by the model, in that:

(i) No cross diffusion of atoms at the Al–Mo interface was observed as no atoms of one element moved into the bulk of the other element;
(ii) At the Al–Mo interface, Al formed a non-wetting layer adopting the morphology of the exposed Mo (100) crystal;
(iii) The calculated mean surface (or interaction) energy at the Al–Mo interface was found to be ~203 mJ/m^2^ (at 1200 K).

**Figure 8:**
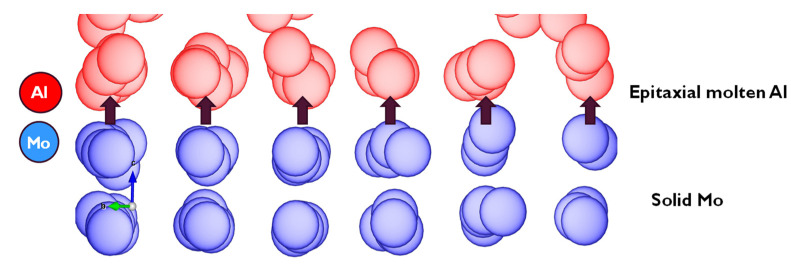
The epitaxial alignment of molten Al to Mo (100) plane.

The non-wetting phenomenon is seen more clearly in Figure 8, where an epitaxial alignment of Al atoms is observed, extending a few layers along the (100) crystal plane, using the exposed metallic Mo (100) crystal morphology as a seed. The small interfacial energy obtained and hence the meagre interaction between the two layers can be categorized as weak van der Waal (or electrostatic) attraction, which further enhances the earlier observation that the Al (100) layer formed at the interface is non-wetting with reference to the interfacial Mo (100) layer. 

**Figure 9:**
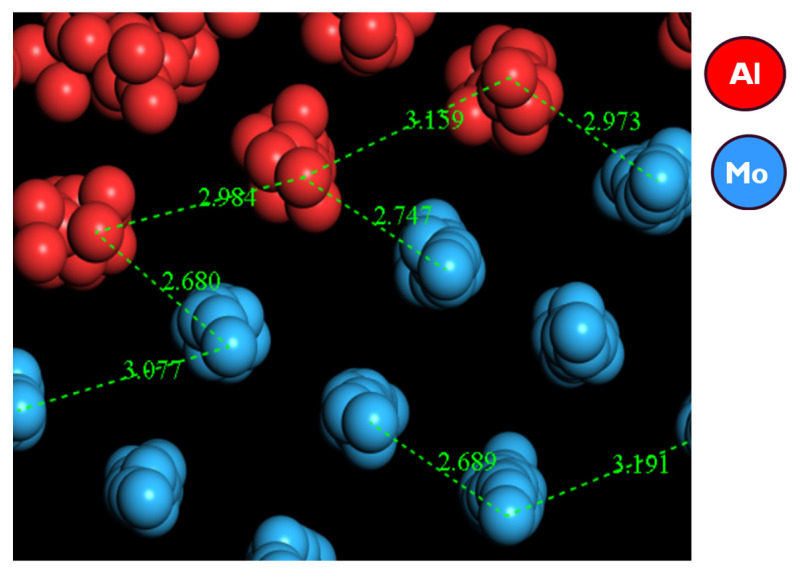
The predicted atomic distances of solid Mo in contact with molten Al.

Data values of Al and Mo bond lengths are summarized in Table 2, and Figure 9 provides sample bond lengths from our modelling, which were found to be within the expected range [39]. This indicates the reliability of the model developed between the metallic Mo (100) layer and the molten Al (100). The major difference in the Al-Al bond length in this work (3.0 Å) and the data figures (of 2.79 Å) is that the larger value predicted in our modelling arises because the Al is in a molten state.

### 3.2. Surface Energy Determination

**Figure 10:**
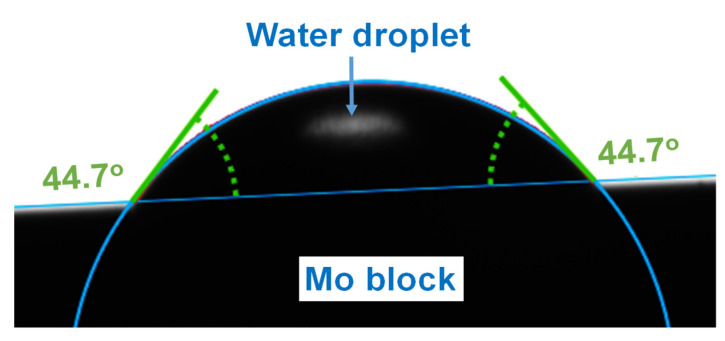
The contact angle measured by the Krüss goniometer using water on a polished Mo block.

Since the contact angle, adhesion, and surface free energy are linked, determination of the contact angle was used to estimate the wettability of the Mo surface. The sessile drop method, which analyzes the drop shape, was used to measure the contact angles between selected liquids and the solid samples. Figure 10 illustrates how the contact angle of the bulk Mo metal was determined to be 44.7° using water. In a similar fashion, the interaction between the Mo surface and formamide or diiodomethane gave contact angles of 23.8° and 37.8°, respectively. 

In this analysis, the instrument was calibrated using polycarbonate as a reference material, and after solving the equations associated with the VCG theory represented by Equations (4) and (5), the surface energy was found to be 53.7 mJ/m^2^ for a polished Mo surface at an ambient temperature. Comparing this with about 203 mJ/m^2^ derived from the classical MD modelling at 1200 K suggests van der Waals interactions between two metals (solid Mo and molten Al). Ordinarily, surface energy should decrease with increasing temperature. Some authors have documented the respective experimental values of the liquid surface energy and the solid surface energy for bulk Mo metal to be 2250 and 3000 mJ/m^2^ [40]. Within the transition metals, the ones that exhibit the largest solid surface energies are among the most catalytically active elements (Re, Os, Ru, etc.). From the theoretical study of low-temperature atomic structures of the Mo (100) plane at equilibrium, the surface energy per unit area was found to be 3340 mJ/m^2^ [41], or ideally 3170 mJ/m^2^, although the (100) plane was deemed to be the least stable [42]. 

In this work, the determined surface energy for the pure Mo–Mo interfacial slabs at 1200 K by Forcite (Materials Studio^®^, Vélizy-Villacoublay, France) modelling was found to be 1542 mJ/m^2^. Other studies indicate that the average surface free energy of solid Mo measured at 1500 °C was 2050 mJ/m^2^ [43], while that determined at 2500 °C was 1900 mJ/m^2^ [44]. Moreover, it has also been observed that the rougher surfaces tend to have larger relaxations and higher surface energies per surface atom [41]. Nevertheless, the major difference between these two sets of surface energies that can easily confuse a reader is that the value in the range of ±2000 mJ/m^2^ is the energy required to break the metallic Mo-Mo in the solid material, while the value of ~54 mJ/m^2^ (modelled by the VCG theory) or 203 mJ/m^2^ (modelled by the Materials Studio^®^) means that the interaction between the solid Mo and the liquid molecules (e.g., H_2_O or molten Al) is dominated by the weak van der Waals forces.

### 3.3. The Static ALCAN Immersion Test

#### 3.3.1. Mass Loss Analysis

**Figure 11:**
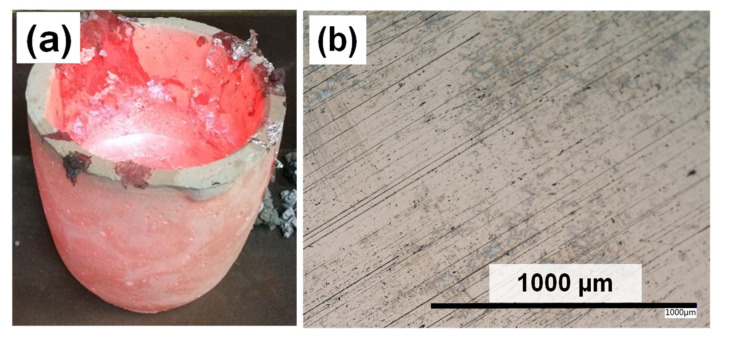
(**a**) A picture of the hot crucible used in the ALCAN immersion test (with size on a reduced scale); and (**b**) an optical image of the Mo-block surface after cleaning with HCl.

Evidence from the ALCAN standard immersion test [45], performed at 850 °C (1123 K), demonstrates the unique anti-wetting properties of a pure Mo-block (99.97%), when immersed in molten Al-5 wt.%Mg alloy, and the corrosion resistance towards Al attack was observed and analyzed [36]. Figure 11a shows a picture of the clay-bonded graphite crucible (used to test the samples in molten Al-Mg alloy) immediately after being removed from the furnace. The photo is on a reduced scale (not provided). Figure 11b shows the surface of the cooled Mo-block from the molten Al-Mg alloy after cleaning with HCl acid. Measurements of the Mo-block taken after the static ALCAN immersion test indicated a decrease of both its mass and size as summarized in Table 3. There was a mass loss of about 20%, accompanied by an 11% drop in the surface area of the cylinder. 

#### 3.3.2. Analysis of Anti-Wetting Properties through Optical Microscopy

**Figure 12:**
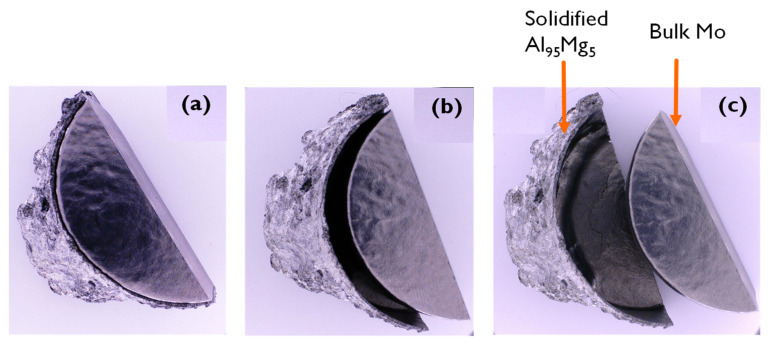
Anti-wetting properties: Images in (**a**), (**b**) and (**c**) show different positions of the Mo-block associated with the solidified Al-Mg alloy to indicate the clean separation of Mo surface from the molten Al-Mg alloy after ALCAN standard immersion test for 96 h.

Optical microscopic images convincingly revealed the anti-wetting properties of Mo in a molten Al-Mg alloy, evidenced from the ALCAN standard immersion test. After cooling, the Al-Mg alloy peeled off easily without sticking to the surface of the Mo sample as depicted in Figure 12. This demonstrated the anti-stick properties of Al on Mo surface.

#### 3.3.3. SEM Analysis at the Mo/Al-Mg Interface

**Figure 13:**
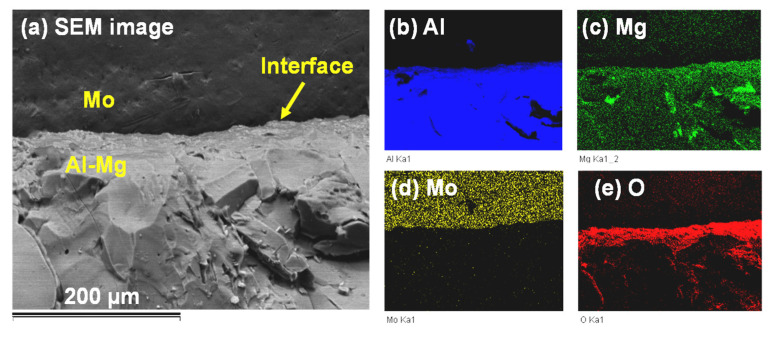
SEM imaging at the interface between the Mo block and the molten Al-Mg alloy after the ALCAN standard immersion test showing (**a**) separation of Mo from the Al-Mg alloy; with EDX elemental mapping for (**b**) Al; (**c**) Mg; (**d**) Mo; and (**e**) O.

Higher-resolution images by SEM analysis indicated that there were no intermediate Mo-Al alloys at the interface between bulk Mo and the Al-Mg alloy. Figure 13a shows the SEM image that reveals a lack of interaction between the bulk Mo and the Al-Mg alloy. There is a close association between Al and Mg in the alloy matrix as indicated by the EDX elemental analysis provided in Figure 13b,c, respectively, while Figure 13d shows complete segregation of Mo from the Al-Mg alloy. The presence of oxygen on the part of the Al-Mg alloy in Figure 13e alludes to the fact that the alloy might have reacted with atmospheric oxygen after the ALCAN immersion test. 

### 3.4. Materials Characterization

#### 3.4.1. Elemental Analysis by OES

Table 4 shows representative elemental-analysis results of the molten Al-Mg samples scooped from the crucible that were analyzed before and after the Mo block was immersed into the Al-5 wt.%Mg alloy at 1123 K. The total mass balance provided in the table does not include many trace elements that were present in the Al–Mg matrix, such as Ba, Ca, Cr, Cu, Ga, Hg, Ni, Pb, Sb, Sn, Sc, V, and Zn, among others, that bring the total to 100%. Overall, it was observed that Mo hardly dissolves into the bulk of the highly corrosive Al-Mg alloy since the total concentration of Mo in the Al-Mg mixture after 96 h was about 0.012% from 0.005%. Since the presence of Mo was not detected in the bulk of the molten Al-Mg alloy as shown by the OES results, it means that the 20% mass loss experienced in the Mo block was due to intergranular corrosion chipping away at the sample.

#### 3.4.2. SEM Analysis

**Figure 14:**
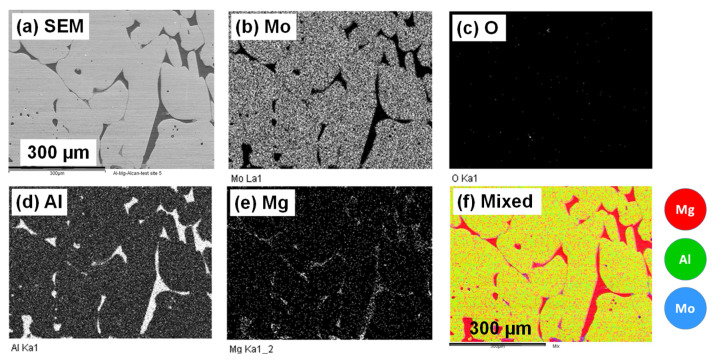
(**a**) SEM imaging on the Al-Mg alloy solidified next to the Mo-block surface, showing bulk Al-Mg (deeper and dark regions), the outer and brighter Mo-Al alloy coating, with EDX elemental mapping for (**b**) Mo, (**c**) O, and (**d**) Al, which is associated with (**e**) Mg, while (**f**) is the mixed color image.

The molten Al-Mg sample that was in contact with the Mo block was analyzed by SEM imaging after the ALCAN immersion test. In principle, SEM images in the backscattered electron mode apply contrast to identify elements by their weight, thus designating the brighter regions to signify the presence of heavier elements of the periodic table in the material, while the darker regions connote lighter elements. Figure 14a is a backscattered electron image (BEI) of the Al-Mg alloy that solidified closest to the Mo-block surface showing two phases. The bulk Al-Mg alloy is represented by the deeper and darker regions of the sample that were encapsulated by the outer and brighter Mo-Al alloy coating, which is suspected to form at the interface between the Mo-block and the molten Al-Mg alloy. The individual EDX elemental mappings are displayed as the brighter regions in Figure 14b for Mo, with none in Figure 14c for O, Figure 14d for Al, and Figure 14e for Mg. The red coloration in Figure 14f confirms the exclusive presence of Mg in the deeper and darker areas of Figure 14e. The brighter regions of Figure 14d,e reveal the association of Mg with Al, implying that the deeper regions of the image shown in Figure 14a are clearly associated with the bulk Al-Mg alloy. 

**Figure 15:**
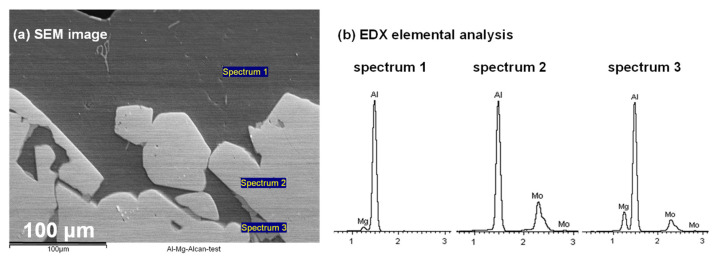
(**a**) The BEI by SEM complemented by (**b**) EDX spectra of the Al-Mg alloy solidified on the Mo block, comprising the deeper bulk Al-Mg-rich areas (spectrum 1); the outer Al-Mo-rich coating (spectrum 2); and the scanty intermediate region with Al-Mg-Mo coating (spectrum 3).

Figure 15a provides the BEI of the Mg-Al alloy in proximity to the Mo-block. EDX spectra for elemental analysis is also shown in Figure 15b, corresponding to spots that represent each unique region sampled. The darker areas dominating the upper side of Figure 15a analyzed by EDX (spectrum 1) indicate the area that exclusively contains the presence of Al and Mg as found in the bulk of molten Al-5 wt.%Mg alloy. The brighter raised regions exemplified in the lower part of Figure 15a analyzed by EDX (spectrum 2) describe the newly formed Al-Mo coating wrapped over the bulk Mo block at its interface with the molten Al-Mg alloy. Analysis by EDX (spectrum 3) points to the scanty Al-Mg-Mo phase with an intermediate color between the two dominant strata: The darker layer beneath, composing an Al-Mg phase, and the elevated (brighter) Al-Mo phase. 

It has been observed that interactions of metallic materials with molten Al possess some common features, as they all form intermediate layers of intermetallic compounds between the metal substrates and molten Al, and these layers usually consist of either one phase or several phases, depending on the composition of the substrate and the reaction conditions [10]. At temperatures above the melting point of Al (660 °C or 933 K), the formation of Al-Mo alloys, which include, among others, both the Al-rich phases (e.g., Al_3_Mo, Al_4_Mo, and Al_5_Mo) and the Mo-rich phases (e.g., AlMo_3_ and Al_8_Mo_3_), are possible examples derived from the Al-Mo phase diagrams [46]. In this work, it was predicted by FactSage™ thermochemical software that an encounter of Mo with molten Al would produce Al_4_Mo, Al_5_Mo, and Al_8_Mo_3_ alloys, with the Al_8_Mo_3_ phase persisting at higher temperatures. However, EDX elemental analysis using SEM imaging did not provide convincing evidence of the kind of phases present. 

#### 3.4.3. XRD Analysis

After the ALCAN immersion test, XRD analysis of the bulk Al–Mg matrix in close proximity to the Mo-block (the side represented by Figure 15) revealed that a reaction had occurred between Mo and molten Al-Mg alloy. The ICDD cards used to identify the materials were [04-010-6160] for Al [47] and [00-042-1120] for bulk Mo [48]. 

**Figure 16:**
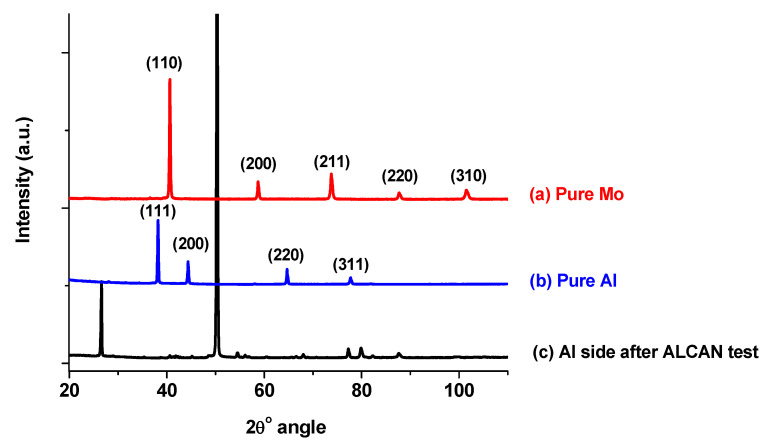
XRD patterns of sample for the bulk (**a**) Mo, (**b**) Al, and (**c**) Al-Mg in close proximity to the Mo block sample after the ALCAN immersion test.

Nevertheless, the chemical composition of the alloy product could not be ascertained, as exemplified in Figure 16, which provides the XRD patterns of the bulk Mo in Figure 16a, bulk Al in Figure 16b, and bulk Al-Mg in Figure 16c. There was no card to match the XRD pattern in Figure 16c, which clearly did not take the form of either pure Mo or Al, and nor were there any possibilities of de-convoluting the peaks Al-Mg-Mo into its various alloys. This means that, potentially, there could have been the co-existence of several Mo-Al alloys at the interface, which were in direct contact with the Mo block. 

## 4. Discussion

The main purpose of this work was to seek ways of lowering the adhesion of molten Al onto materials that are exposed to it. This was achieved by first analyzing the energetics of materials such as Mo metal with low surface energies and use the information to engineer coatings with anti-stick or anti-wetting properties. These materials should also exhibit anti-corrosion characteristics for a prospective application in the Al industry. For example, one potential use of such coatings will be their application on the cast iron rotors used to stir the molten Al-Mg alloy in the ALCAN’s Treatment of Aluminum in Crucible (TAC) process. Since corrosion diminishes the lifespan of the cast iron rotor, it necessitates coating with a material that will slow down the process. Therefore, choosing cast iron as a substrate onto which the deposition of the proposed Mo-based coatings was geared towards its ultimate application in the Al industry.

The originality of this work lies in the blending of various scientific tools, in the use of modelling to predict the anti-wetting properties of Mo in contact with molten Al to establish and experimentally verify the anti-wetting properties of pure Mo. This information may later be used to design, as a benchmark, a pure Mo coating through plasma (SPS) technology, successfully synthesize it, and test it in the Al industry where it has potential applications. The unique choice of a cast-Fe substrate for depositing the coating is in line with its current use as the TAC rotor. Our work reporting on the plasma-synthesized Mo-coatings, in addition to their testing and characterization, are treated in other publications. 

Besides using pure Mo, it is evident from the literature that molybdenizing a material improves its surface hardness and elastic modulus significantly [49]. Some authors have asserted that the addition of elements like Mo and Nb in steel at high thermal treatment temperatures changes its microstructure to favor the formation of carbidic phases that improve their hardness, wear resistance, and tribological behavior [50]. According to the theory, the free energies of NbC and Mo_2_C are −129.2 and −23.5 kJ/mol, respectively, and these elements are added in such low quantities that as hardening materials, high amounts of carbides cause fragility in the steel. 

In addition, it has been claimed that the production of the Mo coating should not be done in an oxygen-rich environment because if MoO_3_ is formed, it will either sublime at 600 °C (873 K) or it will melt into a liquid at temperatures above 795 °C (its melting point, i.e., 1068 K) [51]. This is in fact, within the region where the ALCAN immersion test for our studies was performed (850 °C or 1123 K). Therefore, it could be predicted that should MoO_3_ become part of a coating that will be in direct contact with a molten Al-Mg alloy, the loss of the Mo-oxide during operations (above 1100 K) may create holes and aggravate corrosion and subsequently enhance wear in the coating materials. However, after testing the pure-Mo coatings in contact with the molten Al-Mg alloy, the results were found to be contrary to this assertion, with oxides producing unique needle-like structures; details of these findings will be found in a future publication.

### 4.1. Modelling Material Properties

By evaluating the wettability of a material through the concept of surface free energy and using the spreading behavior rather than just the contact angle, it is possible to explain the specific wetting behavior of a unique material such as Mo [52]. Since higher surface energy causes increased wettability, in our case, the low surface energy values observed on a pure Mo surface (53.7 mJ/m^2^ by VCG modelling) indicate that Mo is potentially a non-wetting material. The advantage of the VCG approach lies in the ability to determine individual surface free energies and relate them, through the appropriate mixing rule (usually a geometric mean), to the interfacial free energies between the two phases [53]. The measured surface free energy of a material indicates the degree of spontaneous interaction between the molten Al and the Mo solid. This entirely depends on the material’s surface characteristics such as the topography, chemical composition, roughness, grain boundaries, and presence of defects, dopants, or contaminants, among others [54]. 

Moreover, modelling the interfacial energy between solid Mo and molten Al by the Materials Studio^®^ confirmed that Mo is indeed a non-wetting material from the observed low-interaction surface energy (203 mJ/m^2^). This demonstrates that at 1200 K, molten Al interacts with solid Mo through the weak van der Waals forces. In order to produce strong adhesive forces, the energy must be higher for the molten Al atoms to strongly adsorb on the Mo surface. If liquid Al atoms were chemically adsorbed on solid Mo, substantial sharing of electrons between the Mo surface and Al would create a covalent or ionic bond, and since chemisorption normally occurs at high temperatures, the energy of adsorption ranges between 200 and 400 kJ/mol [55]. Taking the atomic radius of Mo as 1.40 Å, this would translate to a surface energy distribution in the range of 5394–10,788 mJ/m^2^ in a pure Mo system. On the other hand, physisorption usually does not involve the sharing or transfer of electrons between the two elements in contact and is characterized by an enthalpy change of approximately 20–25 kJ/mole (440–674 mJ/m^2^) or less for a pure Mo surface. In the case under investigation, the energies involved were much lower (203 mJ/m^2^), and this alludes to a potential repulsion of the liquid Al from the Mo surface. 

In practice, one can therefore vary the chemical composition of the Mo surface in order to decrease its surface energy, and this will in turn result in lowering its wettability. Having presented a mass loss of about 20% in 96 h during the ALCAN immersion test, this demonstrates that, potentially, Mo cannot be used alone as a coating, unless as a single crystal. Nonetheless, it can be utilized as an anti-wetting additive to improve the corrosion resistance of other materials by generating new phases that create barriers, or which reduce the surface energy of the coating or lower the diffusion rates of the molten Al-Mg alloy into the coatings. 

### 4.2. The ALCAN Immersion Test and Application in the Al Industry

In this research, the addition of 5% Mg to molten Al was carried out to enhance the corrosive nature of the Al melt in order to effectively examine its effects on Mo-based materials within a short period. Some authors have observed that increasing the Mo content in a coating to 6-wt.% improves its wear resistance to about 3.7 times that of the Mo-free coating, but the best corrosion-resistant material had 2-wt.% in the coating [56]. Since the anti-wetting properties and corrosion resistance of Mo has been established in this work (except for intergranular corrosion), creating composites or alloying it with other materials is foreseeable in order to produce functional Mo-containing coatings applicable to the Al industry. It has been observed that the addition of Mo to, for example, Fe-Cr-B alloys enhances their corrosion resistance in static molten aluminum at 750 °C [12].

We have shown that a high-temperature corrosive attack by a molten Al-Mg alloy on a pure Mo block is by intergranular corrosion, which is dominated by uniform thinning of the sample accompanied with a mass loss of 20% and a decrease in surface area by 11%. In isolated areas, an interface composing a variation of Al-Mo alloys seems to have been created between the two phases (of solid Mo and liquid Al, see coatings in Figure 14 and Figure 15). Although the OES elemental analysis indicated that Mo could not be traced in the bulk molten Al-Mg alloy, it is impossible to conceive the idea that the 20% mass loss remained as the thin coating barriers between the two phases at the {Mo_(s)_–Al_(l)_ interface} shown in Figure 15. The significant mass loss can be explained as a result of the Mo block or its alloys undergoing intergranular corrosion with the chipped particles falling at the bottom of the crucible. The lack of detection of Mo by the OES analysis shows that there is no Mo dissolved in the molten Al-Mg alloy. It has been observed that in many liquid metal–solid systems, the formation of a new compound is preceded by the dissolution of the solid in the liquid [16]. The dissolution of a matrix near impurities or inclusions and clusters located in grain boundaries is more than 2 times larger than when compared to inclusions found inside the grains of the given alloy samples [57]. The initial dissolution rate is usually high, but once a layer of the new compound is formed at the interface, the dissolution rate decreases rapidly. In this work, the dissolution phenomenon was not observed, but rather the chipping away of the Mo block.

Some authors have asserted that degradation of Mo proceeds predominantly through the formation of Al_4_Mo at 700 °C, Al_8_Mo_3_ at 750 °C, and Al_4_Mo and Al_8_Mo_3_ at 800 °C for periods varying from 8 to 120 h [58]. The intermetallic layer was observed to break up as long as it grew, due to thermal stresses dispersed in the molten Al, with the weight-loss of Mo varying linearly with time. There are four significant differences contrasting our findings from theirs:

(i) While their tests were conducted at 800 °C and below, in this work, the static ALCAN immersion test was performed at 850 °C, with only small quantities of the Al-Mo alloys being observed at the Mo_(s)_–{Al-Mg}_(l)_ interface;
(ii) Contrary to their case where the Al-Mo alloys formed were known, in the present work, elemental analysis identifying the type of Al-Mo alloys present at the the Mo_(s)_–{Al-Mg}_(l)_ interface was difficult since the alloys were extremely thin and not cross-sectionally visible at the interface (Figure 13);
(iii) The mass loss of ~20% observed in the Mo block in this work was not principally as a result of the reaction between Mo and the molten Al-Mg alloy, but rather due to intergranular corrosion at the grain boundaries chipping away at the Mo block over time;
(iv) Although modelling by FactSage™ thermochemical software predicted the potential formation of Al_4_Mo, Al_5_Mo, and Al_8_Mo_3_ alloys when molten Al-Mg alloy is in contact with Mo, kinetically these alloys would form very slowly. In addition, if the alloys formed around the Mo are insoluble in the molten Al-Mg alloy, they would create an impenetrable barrier that stops further reaction with Mo, which is beneficial to the process. Since it was not possible to detect Mo in the molten Al–Mg matrix using OES elemental analysis, it confirmed that, in this work, neither Mo nor the Al-Mo alloys dissolved in the molten Al-Mg alloy as was predicted by computer simulations using Classical Molecular Dynamics in the Materials Studio^®^ packages.

## 5. Conclusions

This work has used classical molecular dynamics (CMD) simulation to predict the behaviour of a pure Mo solid in contact with molten Al at 1200 K, which is slightly above the experimental temperature (1123 K), and the model projected that:

- No reaction and no cross diffusion of atoms occurs at the Mo_(s)_–Al_(l)_ interface;
- Molten Al atoms form a non-wetting layer that adopts an epitaxial orientation in alignment with the exposed solid Mo crystal morphology;
- The calculated mean interfacial energy normalized per unit area of the simulated cell was found to be about 203 mJ/m^2^, which implies a weak van der Waals interaction between molten Al and solid Mo.

Surface energy measurements by the sessile drop experiment that employs the van Oss–Chaudhury–Good (VCG) theory established a Mo_(s)_–Al_(l)_ interface energy equivalent to 54 mJ/m^2^, which again confirmed the weak van der Waals interaction between the solid Mo surface and potentially any liquid that may include a molten Al-Mg alloy. In addition, modelling the behavior of atoms at the interface between bulk Mo and the molten mixture of Al-5 wt.%Mg alloy using FactSage™ thermochemical and databases software predicted the potential formation of Al_4_Mo, Al_5_Mo, and Al_8_Mo_3_ alloys, with the Al_8_Mo_3_ phase persisting at higher temperatures above 1123 K. These reactions are thermodynamically possible, but kinetically hindered.

Exceptional anti-wetting properties have been demonstrated by a high-purity (99.97%) Mo block towards a molten Al-Mg alloy through the static ALCAN standard immersion test. The mechanism for the formation and composition of the thin Al-Mo coatings on isolated parts of the Mo block is still not well understood, and since these coatings were wafer-thin and scanty, they were too insignificant to account for the mass loss of ~20% in the Mo block. Rather, the mass loss is construed to have resulted from intergranular corrosion at the grain boundaries, where the crystallite particles would slowly fragment from the sample, particularly so where impurities exist, while the bulk of the grains on the Mo block remains largely unaffected.

Findings on the anti-wetting properties of Mo have led to the proposition of using Mo-based materials to research novel coatings that have potential application in the high-temperature and highly corrosive operations found in the Al industry. Future work will involve the development of such Mo-based coating material using induction suspension plasma-spray (SPS) technology with various inorganic additives to create formulations with improved anti-wetting and anti-corrosion properties.

## Figures and Tables

**Table 1:** Lattice parameters of the Al_(s)_-Al_(l)_ coexistence model before and after melting.

Lattice Parameters	Before Melting	After Melting
a	80.99 Å	64.19 Å
b	80.99 Å	62.00 Å
c	161.98 Å	291.28 Å
α	90°	89.29°
β	90°	100.02°
γ	90°	84.09°

**Table 2:** A comparison of bond lengths between metallic Al and Mo with data figures [39].

Metal Bonds	Bond Lengths (Å)	
	Simulated Values (This Work)	Actual Data Values
Al-Al	3.0	2.79
Al-Mo	2.7	2.76
Mo-Mo	2.7–3.1	2.09–3.27

**Table 3:** Sample materials indicating mass loss (20%) after the ALCAN immersion test.

Sample	Before Test	After Test	Loss (%)
Diameter (mm)	44.28	41.27	6.80%
Height (mm)	25.44	24.78	2.59%
Mass (g)	396.30	315.13	20.48%

**Table 4:** The OES elemental analysis of the molten Al-Mg alloy in the crucible before and after the ALCAN immersion test.

Sampling	Al	Mg	Mo	Mn	Si	Fe	Total *
Before ALCAN test	94.2	4.60	0.005	0.320	0.220	0.160	99.545
After ALCAN test	95.700	2.890	0.012	0.370	0.360	0.160	99.492

* Total omits many other trace elements constituting about 0.5%.

## Data Availability

Data collected in this investigation are not accessible to the public but can be availed from the Université de Sherbrooke upon request.
